# A Lightweight Deep Learning Based Microwave Brain Image Network Model for Brain Tumor Classification Using Reconstructed Microwave Brain (RMB) Images

**DOI:** 10.3390/bios13020238

**Published:** 2023-02-07

**Authors:** Amran Hossain, Mohammad Tariqul Islam, Sharul Kamal Abdul Rahim, Md Atiqur Rahman, Tawsifur Rahman, Haslina Arshad, Amit Khandakar, Mohamed Arslane Ayari, Muhammad E. H. Chowdhury

**Affiliations:** 1 Centre for Advanced Electronic and Communication Engineering, Department of Electrical, Electronic and Systems Engineering, Faculty of Engineering and Built Environment, Universiti Kebangsaan Malaysia, Bangi 43600, Malaysia; 2 Department of Computer Science and Engineering, Dhaka University of Engineering and Technology, Gazipur, Gazipur 1707, Bangladesh; 3 Wireless Communication Centre, University Teknologi Malaysia, Skudai 81310, Malaysia; 4 Department of Electrical Engineering, Qatar University, Doha 2713, Qatar; 5 Institute of IR4.0, Universiti Kebangsaan Malaysia (UKM), Bangi 43600, Malaysia; 6 Department of Civil and Architectural Engineering, Qatar University, Doha 2713, Qatar

**Keywords:** brain tumor classification, RMB image dataset, stacked antenna sensor, deep learning, self-ONN, sensor-based microwave brain imaging system

## Abstract

Computerized brain tumor classification from the reconstructed microwave brain (RMB) images is important for the examination and observation of the development of brain disease. In this paper, an eight-layered lightweight classifier model called microwave brain image network (MBINet) using a self-organized operational neural network (Self-ONN) is proposed to classify the reconstructed microwave brain (RMB) images into six classes. Initially, an experimental antenna sensor-based microwave brain imaging (SMBI) system was implemented, and RMB images were collected to create an image dataset. It consists of a total of 1320 images: 300 images for the non-tumor, 215 images for each single malignant and benign tumor, 200 images for each double benign tumor and double malignant tumor, and 190 images for the single benign and single malignant tumor classes. Then, image resizing and normalization techniques were used for image preprocessing. Thereafter, augmentation techniques were applied to the dataset to make 13,200 training images per fold for 5-fold cross-validation. The MBINet model was trained and achieved accuracy, precision, recall, F1-score, and specificity of 96.97%, 96.93%, 96.85%, 96.83%, and 97.95%, respectively, for six-class classification using original RMB images. The MBINet model was compared with four Self-ONNs, two vanilla CNNs, ResNet50, ResNet101, and DenseNet201 pre-trained models, and showed better classification outcomes (almost 98%). Therefore, the MBINet model can be used for reliably classifying the tumor(s) using RMB images in the SMBI system.

## 1. Introduction

At present, brain tumors are a serious cause of death worldwide. The expansion of abnormal cells that develop inside the brain results in a brain tumor. It causes harm to the brain’s major tissues and develops into cancer. It poses a threat to human life, has a deadly prognosis, and has a significant impact on quality of life. However, due to the unrestrained progression of brain tumors, the possibility of developing brain cancer is increasing day by day. The probability of developing brain cancer is increasing over time as a result of the tumors’ unrestrained growth, and it is the tenth leading cause of death in people [1]. Typically, there are two main categories for brain tumors: (i) benign tumors and (ii) malignant tumors [2,3]. Globally, the incidence of brain tumors is rising alarmingly rapidly. The National Brain Tumor Society (NBTS) estimates that 88,970 Americans were living with a major brain cancer diagnosis in 2022, with 63,040 of those individuals suffering from benign tumors and 25,930 from cancerous tumors [2]. According to the report, the survival rate of the patients is only 36%. The benign tumor is made up of non-cancerous cells with a uniform structure and a consistent shape [4,5,6]. It does not expand to other bodily areas or encroach on nearby tissue. A cancerous tissue of the brain with a heterogeneous composition and an irregular form makes up a malignant tumor [4,5,6]. In contrast to malignant tumors, which grow uncontrollably, benign tumors grow relatively slowly. The invasive properties of the tumors increase the death rate, but early diagnosis, monitoring, categorization, and appropriate examination can lower the mortality rate and raise the survival percentage. Furthermore, automatic brain tumor classification from medical images is important for clinical evaluation and treatment planning of brain cancers. In addition, brain tumor analysis, classification, and detection are severe issues for radiologists and medical doctors. The accurate and timely investigation of brain cancer is imperious for the appropriate treatment of this disease. Brain tumor classification is an important technique in medical imaging applications that classifies specific tumors based on head images. Currently, different types of imaging technologies, including PET (positron emission tomography), MRI (magnetic resonance imaging), ultrasound screening, X-ray screening, and CT (computed tomography), are utilized to diagnose brain tumors in advanced medical facilities [4,7,8,9]. These imaging standards assist medical doctors and radiologists to identify different types of health-related diseases, such as brain cancer. These imaging methods’ major downsides include increased cancer risk due to high dose radioactivity, decreased susceptibility, extreme ionizing of brain cells, high cost, and risk for pregnant women and elderly patients [5,6,7,9,10,11,12]. Further, microwave imaging (MWI) has recently attracted a lot of attention from researchers for medical applications because of its remarkable characteristics, such as non-ionizing radioactivity, penetration capability with low power, non-invasiveness, ionization risk-free for the human body, and cost-effectiveness with a low profile [10,13,14,15,16]. Nowadays, researchers have been using microwave imaging technology to overcome the drawbacks of traditional medical imaging modalities [16,17,18,19,20,21]. Antennas play an important role in microwave brain imaging (MBI) technology, where a single antenna acts as a transmitter and others as receivers. Receivers receive the backscattered biosignals, which are then post-processed by the image reconstruction process. The data is then post-processed using the image generation procedure to produce reconstructed images. Different image reconstruction algorithms have been used in microwave head imaging modalities to detect brain tumors [15,18,20,21,22,23,24,25,26]. However, the main limitations of the developed MBI modalities are (i) noisy, blurry, and low-resolution images created by the system; (ii) identification of the tumor and its location is complicated for a non-expert physician and radiologist; and (iii) difficulty in classifying the tumor in RMB images. In order to overcome such limitations, researchers have been applying deep learning techniques to microwave imaging systems [27,28,29,30,31,32].

Deep learning (DL) is a subdomain of machine learning that uses the convolutional neural network (CNN) model to classify images. The CNN has convolutional layers for feature extraction and densely connected layer(s) for classification. Recently, image classification has become an essential role of medical image analysis, in which deep convolutional neural networks (DCNNs) have been used for the last ten decades. The image classification identifies whether the target object or disease is present or not in the image of the investigation. A fine-tuned DenseNet201 (FT-DenseNet201) deep learning model was developed to classify the MRI tumor images [33]. The model achieved 95% accuracy, but it shows less accuracy when classifying the small-sized tumor images. In another study, a pretrained DenseNet201 (PT-DenseNet201) model was proposed to classify the tumors [34]. It is based on multilevel features and concatenation characteristics that can diagnose the tumor at an early stage. The approach achieved 99.34% testing accuracy, but the precision and specificity scores were only 92% and 83%, respectively. The dual pathway DenseNet (DP-DenseNet) architecture model was proposed in [35] to classify tumors. The architecture was evaluated on the BRATS 2017 MRI dataset. The reported precision, F1 score, and dice score were 85%, 88%, and 89%, respectively. The network model can only classify large-sized tumor-based images but not small-sized tumor images, resulting in comparatively poor classification performances. A deep neural network with a generative adversarial network (DGAN) model was proposed in [36] to classify brain tumors based on MR images. The DGAN model used 64 × 64 sized images as input, and it achieved 93% accuracy. It showed low classification performance due to input image size limitations. A pre-trained Inception-v3 classification model was used to classify brain tumors. The approach employed the concatenation method with the Softmax classification technique to classify tumors from the MRI images. The differential deep convolutional neural network (differential-DCNN) model was presented in [37]. The model was evaluated using 17,600 MRI brain images for the classification of the different types of tumors. As a classification performance, the model achieved 95% sensitivity and 93% specificity. In [38], a conventional multi-pathway CNN (CMP-CNN) architecture was presented for tumor classification by using MRI brain images. The model assessed 3064 images for three types of tumor classification and attained 94% sensitivity. A multi-class tumor image classification by ResNet-50 was proposed in [39]. The model used a global average pooling mechanism to enhance the classification accuracy, but it achieved 97.08% mean accuracy and a 90.02% F1 score. A fine-tuned ResNet101 model was presented in [40] for brain tumor classification. The model used a differential evaluation method and a swarm optimization algorithm to improve classification performance. The classification accuracy achieved by this model was 94.4%. Nevertheless, the model may fail to classify noisy, blurry, and small sized images. However, the pros and cons of existing models are summarized in the following Table 1.

In the last couple of years, deep learning-based transformer architecture has been employed for classifying medical images instead of CNN models. An nn-TransUNet model has been used for MRI medical image segmentation tasks [41]. The model used vision transformers and convolution layers in the encoder for enhancing the segmentation and classification performance. The main benefit of the model is that it reduces time complexity and makes it possible to manually tune the hyperparameters for improved training accuracy, but it requires a large amount of memory and a high-performance GPU to train the model. Another transformer architecture-based model, Vision Transformer (ViT), has been used for classifying images and image recognition [42]. The ViT model used linear projections of flattened patches for image classification. The encoder of the transformer model uses multiheaded self-attention and residual connections in every block for increasing classification performance. A residual vision transformer (ResViT) architecture model has been applied for multimodal medical image classification [43]. In [44], the authors have used a zero-shot learned adversarial transformer (SLATER). The SLATER model combines a deep adversarial network with cross-attention transformers to reduce noise in medical MRI images and enhance image classification performances. It is a suitable network architecture for high-performance MRI image acceleration, but it takes a long time to train the model.

Recently, operational neural networks (ONNs) have been applied as a diverse network model for image analyzing, classification, and processing due to their non-linear properties, low computational complexity, simplicity in structure, and high performances. A self-organized ONN (Self-ONN) model was proposed in [45,46] to classify the biomedical images. It is seen that the Self-ONN model can perform better than conventional CNN models if the model architecture and parameters can be tweaked carefully.

Since all the above-mentioned works used deeper architectures, it is natural that these networks require longer training and inference times and are not suitable for portable device deployment. Therefore, there is a demand to design a lightweight deep learning-based classification model to classify the RMB images with better classification performance. The main contributions of this work are specified below:

1. According to the authors’ knowledge, this is the first study to propose a lightweight classification model called microwave brain image network” (MBINet) to classify RMB tumor images using a new machine learning paradigm called the Self-organized operational neural network (Self-ONN) architecture.
2. The proposed MBINet model is implemented and investigated on the RMB tumor images to classify the brain images into six classes: non-tumor (NT), single benign tumor (BT), single malignant tumor (MT), double benign tumor (BBT), double malignant tumor (MMT), and single benign and single malignant tumor (BMT).
3. The Implementation of a sensor-based microwave brain imaging (SMBI) system with a fabricated tissue-imitating brain phantom model to investigate the imaging performance for generating the RMB tumor image dataset.
4. A new Self-ONN model, MBINet, four other Self-ONN models, two conventional CNN models, and three pretrained models (DenseNet201, ResNet50, and ResNet101) are investigated on the RMB tumor images to classify six classes to show the usefulness of the suggested MBINet classification model.
5. The proposed MBINet model is compared with the seven most recent state-of-the-art models to verify the classification outcomes.

The remaining part of the manuscript is structured as follows: Section 2 explains the SMBI implementation setup and image reconstruction process. The research methodology and materials, including dataset preparation and experimental methods, are discussed in Section 3. Section 4 discusses the results of the classification models for the raw RMB images. In the end, the paper is concluded in Section 5.

## 2. Stacked Antenna Sensor-Based Microwave Brain Imaging (SMBI) System Development and Image Reconstruction Process

### 2.1. Design and Development Process of the Sensor-Based Stacked Antenna

**Figure 1:**
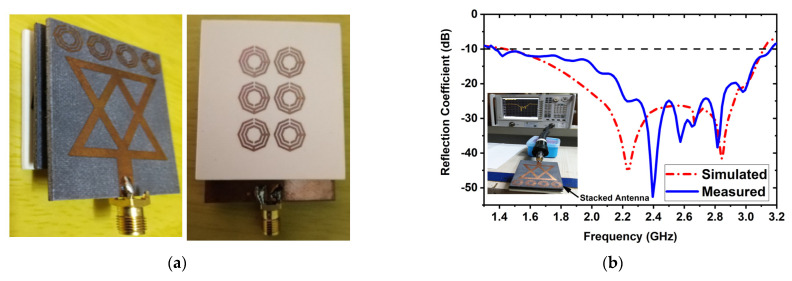
(**a**) Fabricatted 3D stacked antenna, (**b**) Measured and simulated reflection coefficient.

An experimental stacked antenna sensor-based microwave brain imaging (SMBI) system has been developed in this research to reconstruct microwave brain (RMB) images and examine system performance. It is worth mentioning here that a wideband antenna sensor with high gain and unidirectional characteristics is required with a frequency band of 1 GHz to 4 GHz for the MBI system [10,14,17,18,20,21,25,47,48,49]. A new metamaterial (MTM)-inspired 3D wideband stacked antenna sensor has been printed on inexpensive Rogers RO4350B and RT5880 substrate materials. Three (03) substrate layers, together with two air gaps, comprise the antenna sensor. Double-sided foam tape is used to attach the layers together. The bottom layer (BL) is printed on an RO4350B substrate, while the top and middle layers are printed on a RT5880. The air gap in the middle is 2 mm. In the top and middle layers, a single 1 × 4 MTM array component is employed, while a single 3 × 2 MTM array component is employed in the BL. In order to improve antenna performance in terms of effectiveness, realized gain, bandwidth, radiation directionality in open space, and near proximity to the head model, MTM array elements are utilized in layers. The optimized dimension of the antenna sensor is 50 × 40 × 8.66 mm^3^. The antenna was measured in both free space and near the head model to ensure antenna performance. The measurements reveal that the antenna has an appropriate field penetration in the head, a fractional bandwidth (FBW) of 79.20 percent (1.37 to 3.16 GHz), 93 percent radiation efficiency, a 98 percent maximum fidelity factor, and 6.67 dBi gain. The fabricated antenna and reflection coefficient (S-parameters) measurements are shown in Figure 1. These results ensure that the antenna is able to produce the desired RMB images from the implemented SMBI system. We utilized our new MTM loaded 3D wideband stacked antenna in the SMBI system framework to generate RMB images for this research.

### 2.2. Phantom Model Fabrication Process and SMBI System Implementation Process

**Figure 2:**
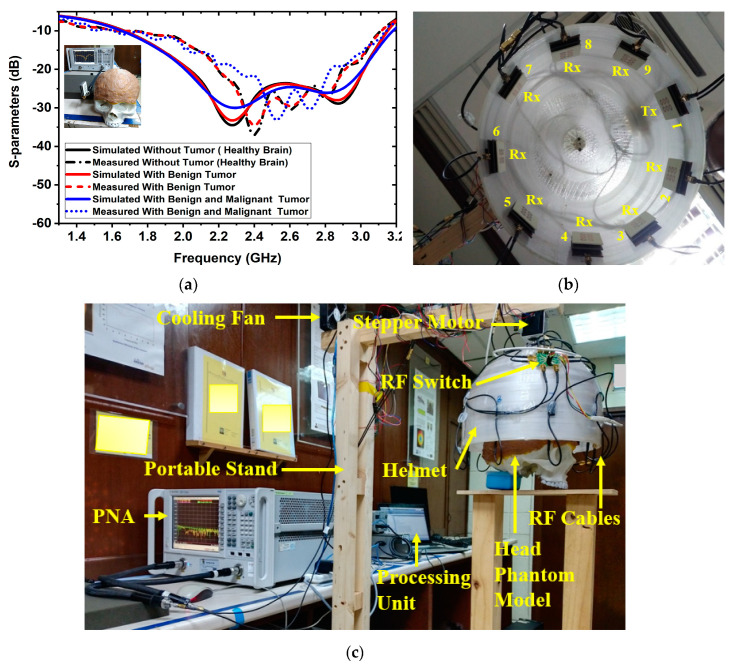
Antenna sensor measurements and experimental setup for the SMBI system: (**a**) Measured and simulated results of the antenna sesnor with a fabricated head phantom model, (**b**) MTM loaded 3D stacked antenna sensor inside the helmet, (**c**) Overall SMBI system model.

At first, a six-layered, tissue-imitating phantom with tumor (benign and malignant) tissues was made for validating the SMBI system. The layers and tumors were constructed as stated in the recipe in [50]. The electrical characteristics of the tumors (i.e., malignant and benign) were deemed to be stated quantities in [51]. The malignant tumor was formed in an irregular elliptical and triangle shape, whereas the benign tumor was created in a roughly round form with a typical shape [51]. Later, layer by layer, the tissue-imitating phantoms and tumors were added to the 3D head model. For the purpose of performing the measurement using the brain imaging equipment, the benign and malignant tumors were put into the model at various locations. The benign and malignant tumors were embedded into the skull model in various places. The simulated and measured S-parameters of the antenna sensor with the formulated tissue-imitating head model, including tumors, are shown in Figure 2a.

Additionally, nine antenna sensor arrays set up inside the helmet are shown in Figure 2b. Figure 2c indicates the complete experimental SMBI system. The system comprised nine stacked antenna sensor arrays, a stepper motor, an adjustable stand, a custom-made helmet, an RF switch, a PNA E8358A transceiver, and a microcontroller. The movable platform, to which the stepper motor is attached, turns counterclockwise with a 7.2° angle at each step to cover the entire (360°) area. The helmet is tightly attached to the motor’s shaft. The diameter of the helmet is 300 mm. Through the use of double-sided foam tape (DSFT), the antenna sensor is secured inside the helmet. In order to cover the entire system, there must be a 40° angle between each antenna. The sensor location is set 100 mm up from the lowest point of the helmet to fine-tune the phantom head position. Through the GPIB port, the PNA is connected to the computer. Port A is linked to the transmitting antenna, while Port B is linked to an RF switch to receive backscattered signals. For assessing the effectiveness of the system, a six-layered 3D phantom model is constructed and mounted in the middle of the helmet. The PNA accumulated the reflected sensor signals (S21, S31, … S91) after each 7.2-degree rotation.

Thereafter, the obtained sensor waves were processed and operated by the iteratively corrected delay-multiply-and-sum (IC-DMAS) image generation algorithm [52] to produce RMB images. It is notable that recently, in vivo imaging technology has been applied in medical science for brain tumor diagnostic purposes. Due to the fact that it is non-invasive, it can visualize living organs (for example, brain tumor imaging) and accurately detect target object locations in the region of interest. However, a live object such as an animal or human body is needed to produce an image, which is very challenging and a medical permission issue. In this research, we cannot use a live brain or human body for generating tumor-based images because of clinical trial limitations in our research lab. As a result, we are unable to collect in vivo image samples. Thus, we used a fabricated tissue-mimicking brain phantom model, which has the actual properties of a real brain, and collected all data from the system. Following that, all of the phantom-based data was analyzed and processed in order to generate brain tumor-based images for further evaluation. However, our future aim is to collect in vivo images for further evaluation, which will help medical doctors classify the tumor easily.

### 2.3. Illustration of RMB Image Samples

**Figure 3:**
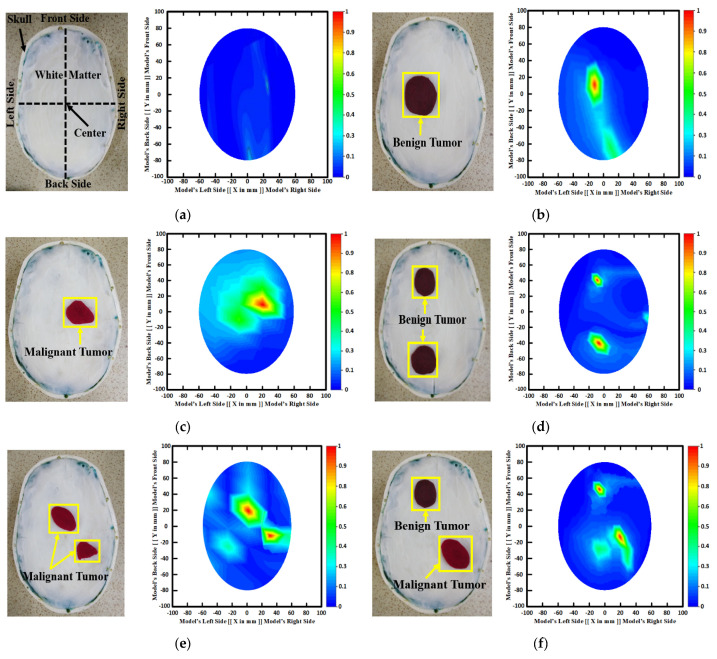
Samples of formulated tissue-imitating head phantom models with RMB images: (**a**) Non-tumor (NT), (**b**) Single BT image, (**c**) Single MT image, (**d**) Two BBT image, (**e**) Two MMT image, (**f**) Single benign and single malignant tumor (BMT).

Figure 3 depicts samples of the tissue-mimicking phantom, different tumor scenarios, and corresponding RMB images. Figure 3a depicts the layout of the phantom model for visual comprehension. In addition, the tumors were positioned at various locations on the head model. Figure 3a–f depict the six image classes: the NT image, a single BT image, a single MT image, a double BBT image, a double MMT image, and a single benign and malignant tumor (BMT) image. After that, we collected nine hundred twenty (920) samples, including all classes, to make the original RMB image dataset. Later, different pre-processing steps were applied to the images to train, validate, and test the classification models. The proposed classifier was investigated by utilizing the original RMB images. Image preprocessing and augmentation processes were applied to the collected image samples to produce a large enough training dataset.

## 3. Methodology and Materials

**Figure 4:**
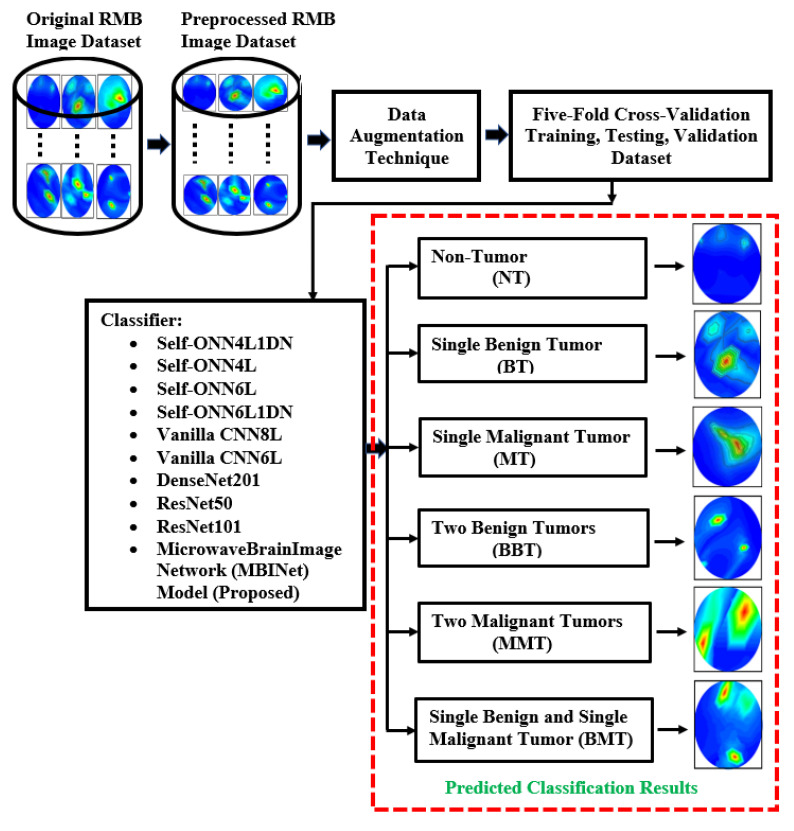
The flowchart for the complete research process.

This segment explains the methodology, dataset clarification, preprocessing method, data augmentation processes, and investigational analysis. The summary of the overall research methodology is stated in Figure 4. This research work utilized reconstructed microwave brain (RMB) images, which were collected from two data sources: (i) this research work (implemented experimental MBI system), and (ii) our previous research work [53] to enrich the dataset for classification models. Generally, the research has primarily focused on two distinct categories of images, such as (i) healthy brain (i.e., non-tumor (NT) images) and (ii) unhealthy brain (i.e., tumor-based images). The unhealthy brain images are categorized into five classifications: (i) single benign tumor (BT) images, (ii) single malignant tumor (MT) images, (iii) two benign tumor (BBT) images, (iv) two malignant tumor (MMT) images, and (v) single benign and single malignant tumor (BMT) images. The work explored the proposed lightweight microwave brain image network (MBINet) classification model; four Self-ONN classification models; two CNN-based models; and two pretrained models were used to inspect the classification performances for the six class classifications: NT, BT, MT, BBT, MMT, and BMT.

### 3.1. Preparation of Image Dataset

**Figure 5:**
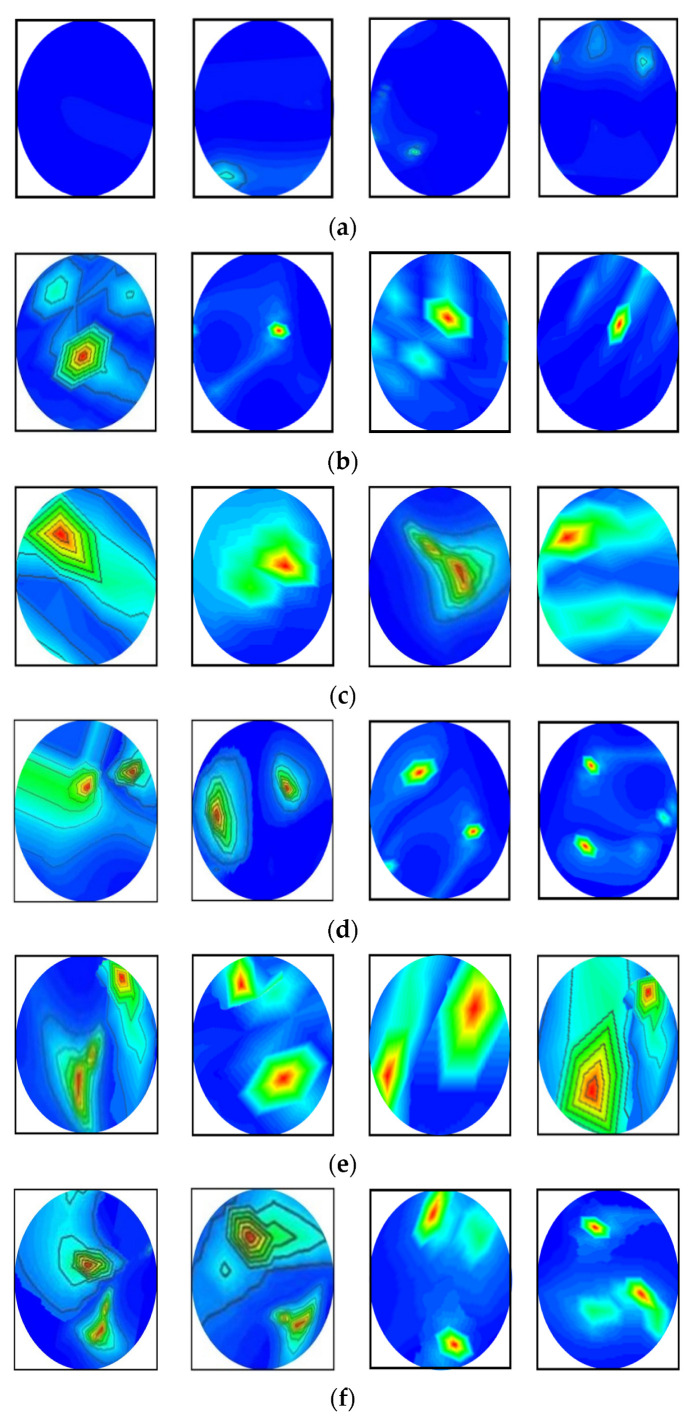
Randomly selected RMB image samples from the original dataset: (**a**) NT class, (**b**) Single BT class, (**c**) Single MT class, (**d**) BBT class, (**e**) MMT class, (**f**) BMT class.

In this research, the dataset is prepared by collecting the image data from two sources: our currently implemented MBI system, and our previous research [53]. The combination of two datasets enriches the training dataset for better classification performance. The dataset consists of a total of 1320 original RMB images, where there are three hundred (300) images for the NT class, two hundred fifteen (215) images for each BT and MT class, two hundred (200) images for each BBT and MMT class, and one hundred ninety (190) images for the BMT class. Some samples of the original RMB images for all classes are displayed in Figure 5. The source code and original RMB image dataset can be found at: https://github.com/Amran038/Microwave-Brain-Image, accessed on 20 November 2022.

### 3.2. Data Preprocessing and Augmentation Process

This section illuminates the data preprocessing and dataset formulation for the experimental deep learning models. The data preprocessing is the starting stage of a DL model because of model’s input constraints. The different classification network models have different input size requirements. Thus, image data is pre-processed (resized and normalized) before training the models. For the raw RMB tumor image classification purposes, the images are resized to 224 × 224 pixels for four Self-ONNs, two vanilla CNNs, two pretrained models (DenseNet201 and ResNet50), and the proposed MBINet models. The original dataset’s images are normalized using the z-score normalization approach, the mean (M), and standard deviation (SD) of each image. Deep learning models typically require a large image dataset to effectively train the model to classify the images.

The image augmentation technique is employed in this study to build a large training dataset because the small dataset is insufficient for training the models. Instead of gathering additional information or samples, image augmentation might improve the performance of the models. It can significantly increase the diversity of data available for training the models as well as create a rich dataset from a small sample image dataset, which helps to enhance network performance. Different types of image augmentation techniques can be used to enrich the training dataset, such as rotation, scaling, translating, horizontal and vertical flipping, zooming, cropping, anatomically guided distortion elimination, etc. In addition, in particular, realistic augmentation based on recent state-of-the-art image synthesis techniques can be considered for image or data augmentation. This augmentation approach can be very useful for multimodal MRI-CT and multi-contrast MRI medical images (i.e., MRI, CT, PET, X-ray mammography, etc.). For example, SynDiff is the adversarial diffusion model, which is used for medical image synthesis and translation purposes [54]. This model is also used for image sampling. A diffusion probabilistic model was used for image synthesis and data scaling as an augmentation process [55]. In data scaling, authors have assumed that an image dataset consists of integers in the range of 0 to 255 as a pixel value, which scales linearly to [−1, 1] for reducing the data dimensionality [55].

Furthermore, in our microwave imaging technology, the produced images are two-dimensional and almost noiseless, and the target object’s (i.e., tumor) perceptibility is good. As a result, anatomically guided distortion and adversarial diffusion augmentation processes are not needed to apply to RMB images. For this reason, four different image augmentation techniques (e.g., rotation, scaling, translating, and flipping) were used in this investigation to generate the training dataset.

**Figure 6:**
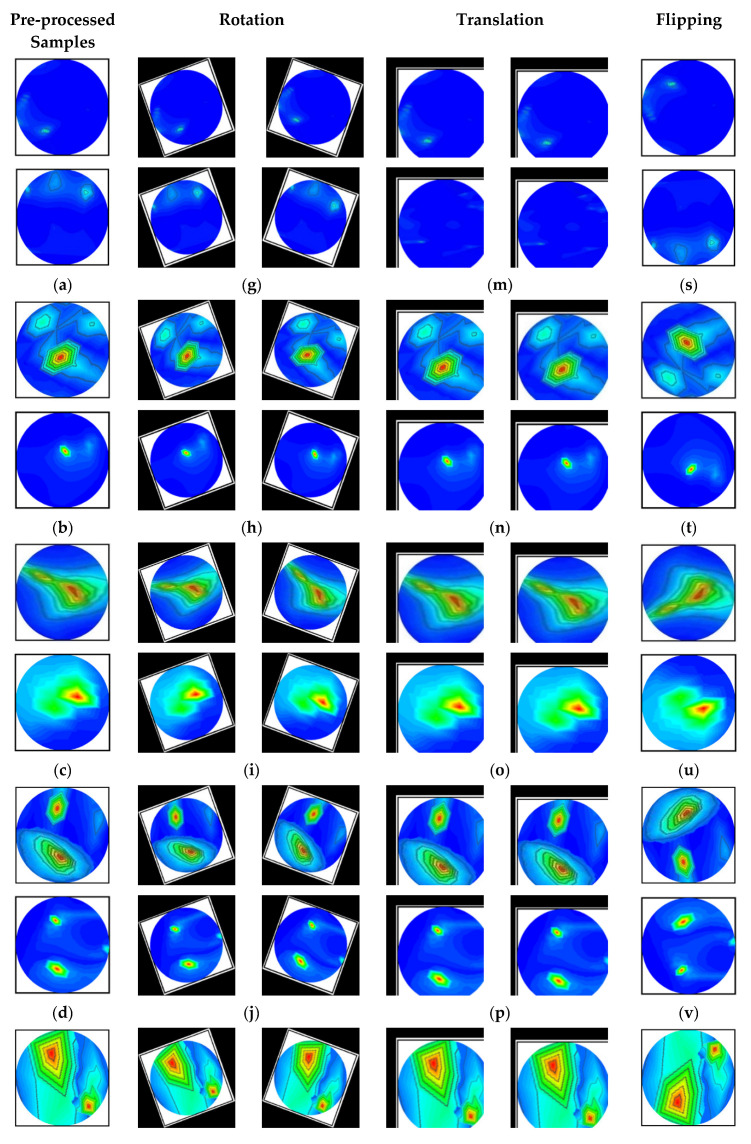

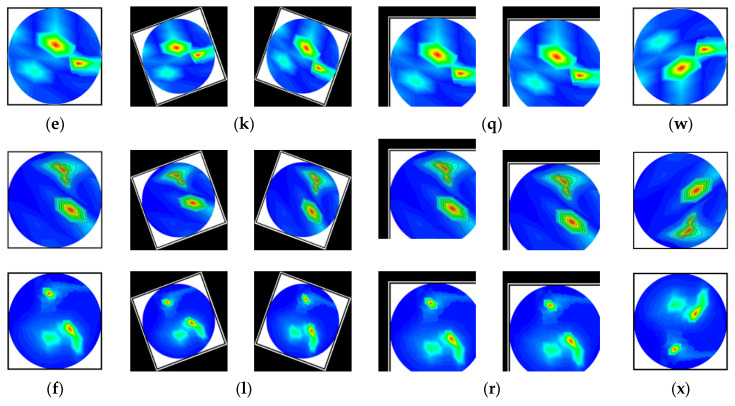
Samples of the augmented training set (six classes): (**a**–**f**) Pre-processed original images, (**g**–**l**) After rotating all class images by 30 degrees both clockwise and counterclockwise, (**m**–**r**) All class images after 10% horizontal and vertical, and 8% horizontal and 12% vertical translation, and (**s**–**x**) All class images after vertical flipping.

The images are rotated in both clockwise and counterclockwise directions at an angle ranging from 10 to 40 degrees. The tumor objects are thus relocated at various locations within the images. Scaling is the process of reducing or enlarging an image. Here, 10% to 12% image magnifications are employed. The image translation technique shifts the tumor objects to different locations in the images by translating the images by 10% to 15% in both the vertical and horizontal directions. The vertical flipping method is also used as an augmentation technique. This study used a five-fold cross-validation technique for training, validation, and testing purposes. Eighty percent of the total images were utilized for training, and twenty percent were used for testing in order to do five-fold cross-validation. Additionally, 20% of the training dataset, which comprises 80 percent of the dataset, is used for validation to prevent overfitting. After augmentation, 13,200 images were created for training, 264 images for testing, and 231 images for validation per fold. The complete dataset explanation is shown in Table 2. However, after pre-processing and augmentation, samples of the augmented images for all classes are demonstrated in Figure 6.

### 3.3. Experiments

This study uses the PyTorch library with the Python 3.7 version to construct and run nine alternative classification models, including the proposed MBINet model, on the Anaconda distribution platform. The tests are carried out on a 64-bit version of Windows 10 with 128 GB of RAM and a 3.30 GHz 64-bit Intel(R) Xeon(R) W-2016 processor. Additionally, the network training performance is accelerated using an 11 GB NVIDIA GeForce GTX 1080Ti GPU. The classification performance metrics of the five folds were calculated.

### 3.4. Proposed Microwave Brain Image Network (MBINet) Model—Brain Tumor Classification Model

Recently, an operational neural network (ONN)-based model was established in [56] to overcome the linear nature of the CNN. The ONN is a heterogeneous network that has demonstrated promising performance in a number of applications (image denoising, image restoration, and image classification) [46,57]. It uses a fixed set of nonlinear operators to learn complicated patterns from any input. However, the fixed set of operator libraries restricts ONN’s ability to learn. Self-ONN (Self-organized ONN) is employed to address this issue [58]. Instead of a fixed collection of operator libraries, Self-ONN inevitably discovers the best set of operators throughout the training phase. This develops a more vigorous model that can carry out a wider range of events and generalize effectively in practical situations. Self-ONN networks choose the best set of operators during the training process, which can be a combination of any standard function or some other function that we do not know. The output $O_{k}^{L}$ at kth neuron of Lth layer of any ONN can be illustrated as follows:

(1) $$O_{k}^{L}=b_{k}^{L}+\sum_{i=1}^{N_{L-1}}\Psi_{ki}^{L}\left(w_{ki}^{L},y_{i}^{L-1}\right)$$

where, $b_{k}^{L}$ and $w_{ki}^{L}$ are the weights and biases connected to that layer and neuron, $y_{i}^{L-1}$ is the input from the previous layer, $N_{L-1}$ is the kernel size of layers, $\Psi_{ki}^{L}$ is the nodal operator of neurons and layers. If $\Psi_{ki}^{L}$ is linear than the equation simply corresponds to conventional CNN. In ONN, the combined operator Ψ can be formed by a set of standard functions as follows:

(2) $$\Psi \left(w,y\right)=w_{1}sin(w_{2}y)+w_{3}exp(w_{4}y)+\ldots \ldots +w_{q}y$$

Here, w denotes the *q*-dimensional array of parameters, which is composed of internal parameters of the individual functions and weights. The combined nodal operator Ψ can be built using a Taylor series function rather than a predefined set of operators. The Taylor series is a function f(x), near point, x=a is stated by the following equation:

(3) $$f\left(x\right)=f\left(a\right)+\frac{f^{\prime }\left(a\right)}{1!}\left(x-a\right)+\frac{f^{\prime \prime }\left(a\right)}{2!}{\left(x-a\right)}^{2}+\frac{f^{\prime \prime \prime }\left(a\right)}{3!}{\left(x-a\right)}^{3}+\ldots \ldots +\frac{f^{n}\left(a\right)}{n!}{\left(x-a\right)}^{n}$$

The Equation (3) can be used to construct the nodal operator as follows:

(4) $$\Psi \left(w,y\right)=w_{0}+w_{1}\left(y-a\right)+w_{2}{\left(y-a\right)}^{2}+\ldots \ldots +w_{q}{\left(y-a\right)}^{q}$$

Here, $w_{q}=\frac{f^{\left(n\right)}\left(a\right)}{q!}$ denotes the qth parameter of the qth order polynomial. In Self-ONN, *Tanh* has been employed as an activation function that has a [−1, 1] range-boundary. So, for *tanh*, a is equal to zero in Equation (4).

**Figure 7:**
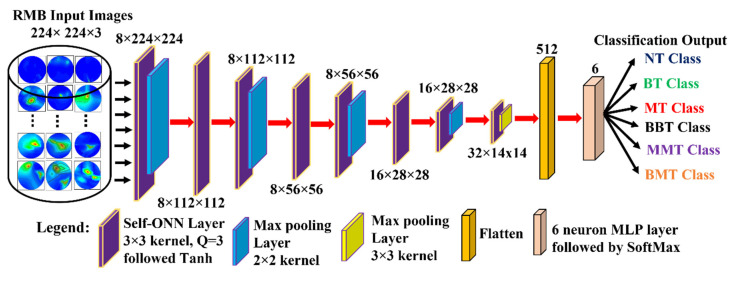
Proposed Microwave Brain Image Network (MBINet) model using Self-ONN.

We developed a new lightweight classification model called microwave brain image Network (MBINet) for classifying the RMB tumor images into six classes: (i) NT, (ii) BT, (iii) MT, (iv) BBT, (v) MMT, and (vi) BMT. The MBINet is constructed by utilizing a self-organized operational neural network (Self-ONN) architecture. The overall architecture of the MBINet classification model is depicted in Figure 7. From Figure 7, it is observed that the MBINet has a total of eight layers, including seven Self-ONN layers and one MLP (multilayer perception) layer. In the architecture, the first five layers have eight neurons, two have 16 neurons, and one has 32 neurons, respectively. Through the self-organization of its nodal operators, it can accomplish the required non-linear transformations to extract optimal features from the brain tumor images. The kernel sizes are set to ×3 for all Self-ONN layers. The kernel size of max pooling layers is set to 2 × 2 for the 1st, 3rd, 5th, and 7th layers, and 3 × 3 for the 8th layer to make it lightweight. Moreover, the Q value is set to 3 as the order of qth order polynomial for all operational layers. The input image dimension is set to 224 × 224 with 3 channels that are fed to the input layer of the model. Images are propagated through the Self-ONN and Max polling layers, and features are extracted into various feature maps. A flattening layer with 512 neurons is used to convert the output of the convolutional layer into a one-dimensional feature vector and apply it to the final MLP layer. The MLP layer is the final classifier of the network. It uses six neuron layers followed by the SoftMax activation function to classify the upcoming images into six classes: NT, BT, MT, BBT, MMT, and BMT classes.

#### Experimental Analysis of the Classification Models

In this section, we discuss six classification experiments to investigate the classification performances of the proposed MBINet by using the RMB images. However, the proposed MBINet model and four variations of the Self-ONN-based model, such as two Self-ONN models with 4 operational layers and two with 6 operational layers (Self-ONN4L1DN, Self-ONN4L, Self-ONN6L, and Self-ONN6L1DN), as well as two vanilla CNN models with 6 and 8 layers (Vanilla CNN6L and Vanilla CNN8L), and two pretrained models (DenseNet201 and ResNet50), were investigated and the results were compared separately using the raw RMB tumor images. In the model names, “4L” means the model consists of four layers, “6L” means the model consists of six layers, and “1DN” means the model consists of one dense layer in the final stage. The training was executed using a learning rate (LR) of 0.0005 for a maximum of 30 epochs, a batch size of 16, utilized the Adam optimizer for network optimization, and set stop criteria based on training loss. The Q order value is a significant factor during training the models; Q = 1 is set to train the two vanilla CNNs and the three pretrained models, and Q = 3 is set for the Self-ONN and MBINet models. The hyperparameters for the classification models are presented in Table 3.

**Figure 8:**
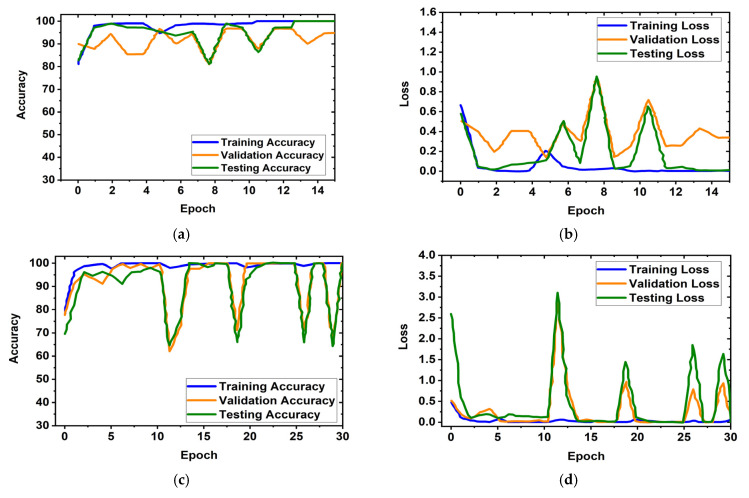

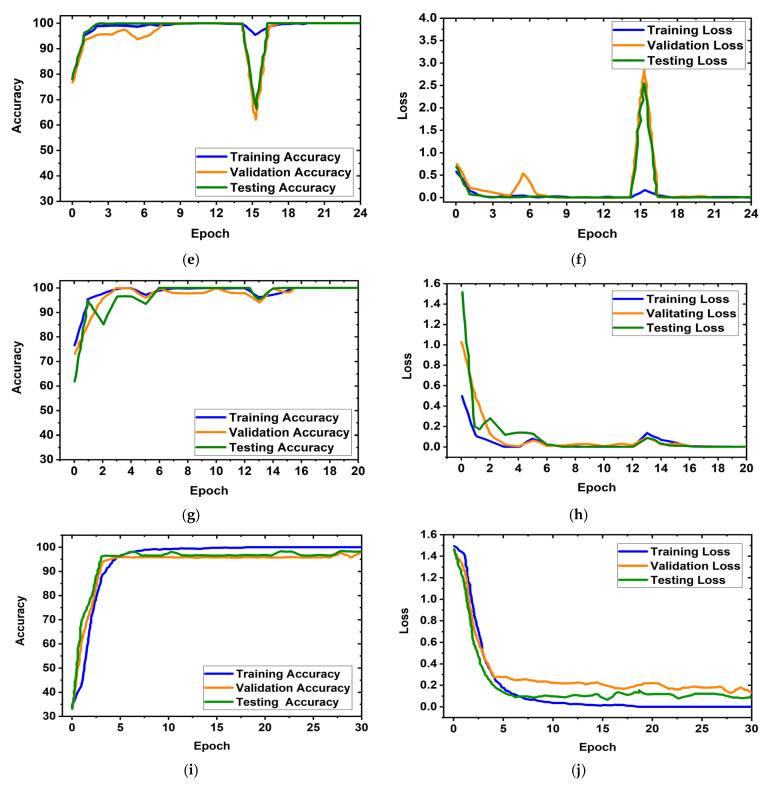
Training, validation, and testing accuracy and loss graphs for five-fold cross-validation dataset: (**a**,**b**) Fold-1, (**c**,**d**) Fold-2, (**e**,**f**) Fold-3, (**g**,**h**) Fold-4, (**i**,**j**) Fold-5.

It is obviously true that overfitting is a major issue in machine learning models, which degrades the performance of the model. Overfitting occurs when the selected model fits more data than is required and tries to capture every piece of data fed to the model. Hence, the model starts capturing noise, imbalance, and inaccurate data or images from the dataset, which reduces its performance and shows high variance and low bias. In order to avoid overfitting problems, four criteria were used in the proposed model: (i) the cross-validation method, (ii) training with more data samples, (iii) stop criteria based on validation loss, and (iv) epochs of patience. In this experiment, a five-fold cross-validation dataset was used, along with random shuffling of the dataset. Following that, the dataset is split using five-fold stratified cross-validation for training, validation, and testing. Eighty percent of the total images were utilized for training, and twenty percent were used for testing in order to do five-fold cross-validation. Additionally, 20% of the training dataset, which comprises 80 percent of the dataset, is used for validation purposes. As a result, the dataset was more generalized, which reduced the biasedness of the model during training and testing result evaluation. If validation loss remained constant after 5 epochs of training, the training process was terminated. Otherwise, training of the model continued up to 30 epochs. Then, the fold-wise performance of the MBINet model was observed. The training, validation, and testing accuracy and loss plots for five folds with respect to epochs of the proposed MBINet model are illustrated in Figure 8. It is seen from Figure 8a that when the model was trained with the Fold-1 dataset, it got saturated after 10 epochs and stopped training after 15 epochs. However, the model achieved very low testing and validation accuracy. On the other hand, the model achieved high testing and validation losses (Figure 8b), but was not overfit. Moreover, when the model was trained with the Fold-2, Fold-3, and Fold-4 datasets, it showed that the testing and validation accuracy gradually increased, and the corresponding losses gradually decreased. Additionally, when the model trained with the Fold-5 dataset, it achieved high training, validation, and testing accuracy with low losses, which are shown in Figure 8i,j. In Fold-5, the model performance gets saturated after 5 epochs, and the model is not overfitted and converges well. Furthermore, the model tried to capture every image fed to it to enhance the classification performance. As a result, the training, testing, and validation accuracy and corresponding losses are almost similar, which ensures the better performance of the model. It is notable that the data splits may permit an unbiased evaluation of the model. As a result, the proposed model is able to classify the RMB images reliably with high classification performance.

### 3.5. Evaluation Matrix for the Classification Model

The classification performance of the various CNN and Self-ONN models is assessed by the five assessment matrices, such as: (i) overall accuracy (A), (ii) weighted recall or sensitivity (R), (iii) weighted specificity (S), (iv) weighted precision (P), and (v) weighted F1-score (Fs). The evaluation metrics are computed by applying the subsequent equations:

(5) $$A=\frac{\left(N_{TP}+N_{TN}\right)}{\left(N_{TP}+N_{FN}\right)+\left(N_{FP}+N_{TN}\right)}$$

(6) $$R=\frac{N_{TP}}{\left(N_{TP}+N_{FN}\right)}$$

(7) $$S=\frac{N_{TN}}{\left(N_{FP}+N_{TN}\right)}$$

(8) $$P=\frac{N_{TP}}{\left(N_{TP}+N_{FP}\right)}$$

(9) $$F_{s}=\frac{\left(2\times N_{TP}\right)}{\left(2\times N_{TP}+N_{FN}+N_{FP}\right)}$$

where *N_TP_* denotes the number of tumor samples detected as tumors, *N_TN_* represents the number of non-tumor image samples recognized as non-tumors, *N_FP_* denotes the number of samples incorrectly recognized as tumors, *N_FN_* denotes the number of samples with tumors that were missed by the network.

## 4. Results and Discussion

### 4.1. Raw RMB Image Classification Performances

In this study, the main advantages of the MBINet model are: (i) a lightweight architecture that uses non-linear operations to increase network diversity as well as classification effectiveness; (ii) the ability to optimize the learning weight of each layer during the training process; and (iii) the ability to achieve superior classification performances while significantly reducing computational complexity compared to conventional CNN models. This section discusses the four Self-ONNs (Self-ONN4L1DN, Self-ONN4L, Self-ONN6L, and Self-ONN6L1DN), two vanilla CNNs (vanilla CNN6L and vanilla CNN8L), three pretrained models (DenseNet201, ResNet50, and ResNet101), and proposed MBINet classification models to investigate the classification effectiveness by applying the original RMB images. The classification models can classify the images into six classes: non-tumor (NT), single benign tumor (BT), single malignant tumor (MT), two benign tumors (BBT), two malignant tumors (MMT), and single benign and single malignant (BMT) tumor classes.

In addition, all classification models were trained using the raw RMB brain tumor images. The comparative classification performance outcomes of the models for the raw RMB brain tumor images are presented in Table 4. It was found that conventional deeper CNN networks have achieved lower performances than the four Self-ONNs models, but the proposed MBINet model was the best model among all networks and achieved the highest performances. The MBINet has exhibited accuracy, precision, recall, specificity, and an F1 score of 96.97%, 96.93%, 96.85%, 97.95%, and 96.83%, respectively, for the raw RMB brain images. In addition, the mean (M) and standard deviations (STD) were considered for quantitative assessments. Further, in these assessments, the test dataset was split into five folds, where every fold consists of 264 images. The calculated average M and STD for five folds are presented in Table 5. It is noted from Table 5 that the proposed model showed lower STD values than other models, which means there was very little variance. Therefore, it is concluded that the proposed classification model exhibited better performance than the other models for classifying the RMB brain images into six classes.

### 4.2. Receiver Operating Characteristics (ROC) Analysis

**Figure 9:**
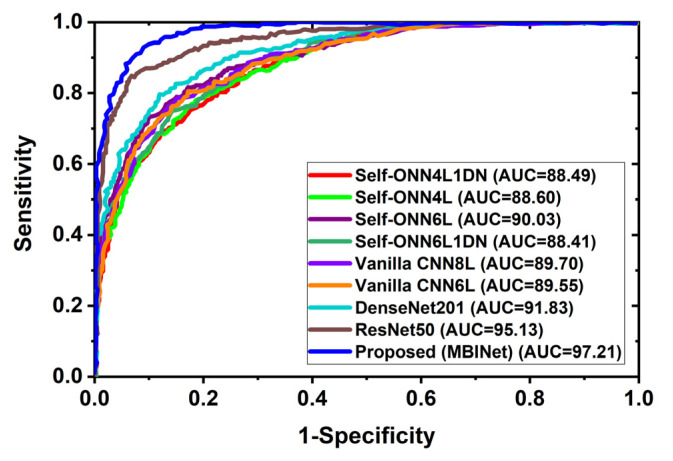
Receiver Operating Characteristics (ROC) curve with AUC.

In multi-class classification issues, the ROC curve is a crucial evaluation metric. A classification model’s performance across all thresholds can be seen using the ROC curve. Additionally, it demonstrates the ability to distinguish across classes. Figure 9 depicts the classification ROC and area under the curve (AUC) for all classification models across all thresholds. Figure 9 exhibited the ROC with AUC for raw RMB image classification and showed that the proposed MBINet model performed better. The computed AUCs for Self-ONN4L1DN, Self-ONN4L, Self-ONN6L, Self-ONN6L1DN, vanilla CNN8L, vanilla CNN6L, DenseNet201, ResNet50, and the proposed MBINet are 88.49%, 88.60%, 90.03%, 88.41%, 89.70%, 89.55%, 91.83%, 95.13%, and 97.21%, respectively. It is observed that the MBINet model performed better than other state-of-the-art models, and it can be reliable for RMB image classification.

### 4.3. Performance Analysis

**Figure 10:**
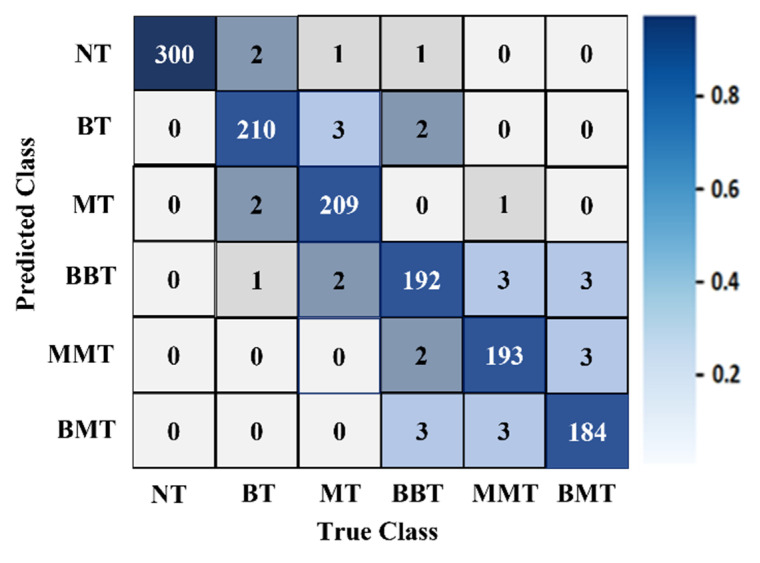
The confusion matrix of the proposed MBINet classification model for the raw RMB brain images.

**Figure 11:**
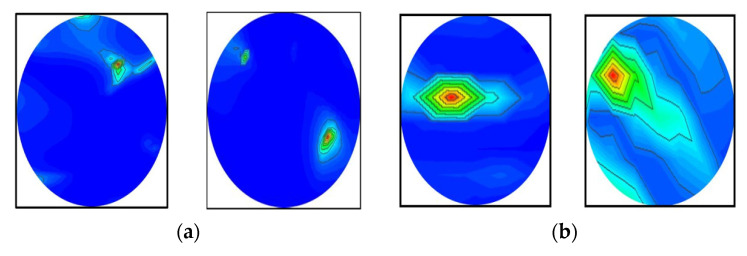

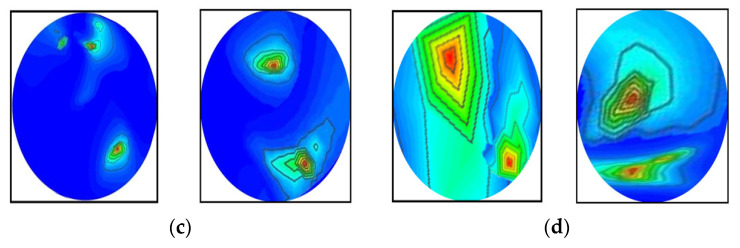
Some misclassified image samples by the MBINet model for the raw RMB images: (**a**) BT images were misclassified as a NT class, (**b**) MT images were misclassified as a BT class, (**c**) BBT images were misclassified as a NT class, and (**d**) MMT images were misclassified as a BBT class.

It was determined from the classification performances in Table 4 and Table 5 that the best classification model was MBINet for classifying the RMB images. The overall classification accuracy was 96.97% for the raw images. For classification results, the confusion matrix of the MBINet model for the raw RMB brain images is illustrated in Figure 10. It is observed from the confusion matrix that the model has been classified at 100%, 97.67%, 97.20%, 96.00%, 96.50%, and 96.84% for NT, BT, MT, BBT, MMT, and BMT classification. It is illustrated that a total of 32 images were misclassified out of 920 images during the testing of the model. For instance, eight misclassified images from four classes (BT, MT, BBT, and MMT) are illuminated in Figure 11.

Figure 10 shows that the BT images were incorrectly classified as NT, MT, and BBT classes; the MT images were incorrectly classified as NT, BT, MMT, and BBT classes; and the MMT images were incorrectly classified as MT, BBT, and BMT classes. In addition, the misclassification rate was comparatively low for the proposed model. However, through the training of Self-ONNs, the optimum non-linear parameters can be learned to exploit the learning performance and attain a superior classification performance in terms of non-tumor and tumor images. Finally, the comparison outcomes of the proposed model with the existing models by applying the same dataset (i.e., the experimental image dataset) are presented in Table 6. The performance metrics: Accuracy (Acc.), Precision (Prc.), Recall (Rec.), Specificity (Spec.), F1-score (Fs), and Overall Classification Performance (OCP) of existing models were calculated and presented in Table 6. It is observed from Table 6 that the proposed MBINet model performed better and showed satisfactory outcomes than other models for the RMB tumor image classification. Finally, it is concluded that the MBINet classification model improved the classification performance and is applicable in the SMBI system for classifying the RMB tumor images into six classes.

## 5. Conclusions and Future Directions

This paper presents the brain tumor classification from the RMB tumor images through a lightweight, deep learning-based microwave brain image network (MBINet) model. The MBINet is based on a self-organized operational neural network. In the beginning, a compact 3D stacked wideband nine antenna array sensor was utilized to implement the SMBI system that produced reconstructed microwave brain (RMB) images, and then 920 raw RMB image samples were collected. In addition, another RMB dataset was collected from our previous work to enrich the training dataset. Later, a lightweight microwave brain image network (MBINet) classifier model was applied to classify the raw RMB images into six classes (NT, BT, MT, BBT, MMT, and BMT). MBINet uses non-linear operations to boost network diversity, increase computational effectiveness, and attain superior classification performance. Furthermore, the MBINet, four Self-ONN classification models, two conventional CNN models, and three pretrained models were examined using the original RMB images, and the classification outcomes were compared. Compared with the state-of-the-art models, the proposed MBINet classification model performed better. The achieved accuracy, precision, recall, specificity, and F1 score of the MBINet model are 96.97%, 96.93%, 96.85%, 97.95%, and 96.83%, respectively, for six class classifications using the raw RMB images. The MBINet model showed better classification results than other models. Further, it is concluded that the MBINet model can be used for consistently classifying the brain tumor(s) from the RMB images and can be utilized in the SMBI system.

### 5.1. Research Shortfalls and Future Improvement

We used the M-DMAS image reconstruction algorithm in this study, which can only reconstruct non-tumor images and two tumor-based images, which is one of the algorithm’s shortfalls. However, if more than two tumors or any other types of tumors, such as meningiomas, pituitary adenomas, craniopharyngiomas, etc., are formed in the brain, the algorithm will not reconstruct the images. On the other hand, in the proposed classification model, the learning outcomes of the MBINet depend on the nodal operators and Q-order parameter values, which must be fixed in advance, which is another shortcoming of the model. In other words, if the right operator setting for proper learning is lacking, the learning outcomes will decrease. Moreover, there is an inadequate discrepancy due to the usage of one nodal operator set for every one of the neurons in a hidden layer. Keeping in mind the mentioned limitations, we can focus on improving the following for our future work: (i) Implementation of a new image reconstruction algorithm that will reconstruct different types of tumors with high-resolution images; (ii) Proper ingredient selection and quantity for fabricating the different types of tumors; (iii) Assessment of the classification performance of the proposed model for classifying different types of tumors by optimizing learning parameters and Q-order.

### 5.2. Future Directions

Based on our evaluations of the research that has already been done, we can suggest a few directions for future research: (i) There is an opportunity to adapt a better optimization algorithm (i.e., AdaGad [59], SGD [60], Adam [61], RMSProp [62]) for training, which should be a modified MBINet model for proper functioning, (ii) Determine an optimal Q-order value that can be automatically used in layers for enhancing the classification performance instead of fixing the Q-value; (iii) Computational complexity is the crucial issue for the Self-ONN model, so finding a computational complexity and inference time reduction mechanism is another research opportunity; and (iv) The model can be assessed by using a large multi-modal or 3D microwave brain image dataset.

## Figures and Tables

**Table 1:** Summarizes the pros and cons of the existing models mentioned in the literature review.

Ref.	Name of the Existing Model	Pros	Cons
[33]	FT-DenseNet201	The Entropy Kurtosis based technique is used in this model for feature extraction to improve the accuracy of the system and reduce the time of classification.	The fusion process of the model increases the computational time and shows less accuracy in classifying the small-sized tumor images.
[34]	PT-DenseNet201	The multi-level features Information is extracted from different bottom layers of the model, which enhances its capability to classify the tumors.	It requires a large amount of computational time and shows a low precision score and specificity score for noisy images.
[35]	DP-DenseNet	The model is a combination of residual networks and dilated convolutional layers that can solve the vanishing gradient problem. It enhances image resolution and classification accuracy for multimodal brain tumor samples.	The network model can only classify large-sized tumor-based images but not small-sized tumor images, resulting in comparatively poor classification performances. Further, it increases the computational time.
[37]	Differential-DCNN	The model uses a differential operator with contrast calculation for analyzing the pixel-directional pattern of images. It is very good at accurately classifying a large set of tumor images.	The model may fail to achieve high performance due to large dataset constraints, which increases the testing loss. As a result, the model failed to classify the small-sized tumor images.
[38]	CMP-CNN	The CMP-CNN model employs three convolutional pathways for extracting discriminant texture features of different kinds of tumors and uses a multi-scale processing strategy for improving the tumor classification performance.	It takes a long computational time to train the model and has caused false positives in a number of testing images due to the lack of variability among the three tumor types.
[39]	ResNet-50	The global average pooling method is used to solve the problems of vanishing gradient and overfitting in the deep network-based ResNet-50 model.	It is computationally expensive as it uses a deep neural network. The classification accuracy is slightly lesser when compared to other classes.
[40]	Fine-tuned ResNet101	The model utilizes the differential evaluation method and particle swarm optimization algorithms to reduce redundant features and computational overhead.	The computational time increases during the testing process because of the fusion process, which achieves less accuracy in classifying the small tumor images.

**Table 2:** Description of the training, testing, and validation datasets.

Dataset	Number of Original Images	Image Classes	Training Dataset					
			Number of Images Per Class			Augmented Train Images Per Fold	Testing Images Per Fold	Validation Image Per Fold
			This Work	Ref. [53]	Total			
Raw RMB Image Samples	1320	Non-Tumor (NT)	200	100	300	3000	60	48
		Single Benign Tumor (BT)	140	75	215	2150	43	35
		Single Malignant Tumor (MT)	140	75	215	2150	43	35
		Two Benign Tumors (BBT)	150	50	200	2000	40	32
		Two Malignant Tumors (MMT)	150	50	200	2000	40	32
		Single Benign and Single Malignant Tumor (BMT)	140	50	190	1900	38	31
Total			920	400	1320	13,200	264	213

**Table 3:** Hyperparameters for all classification models.

Parameter’s Name	Assigned Value	Parameter’s Name	Assigned Value
Input Channels for Color Image	3	Q order	1 for CNN, 3 for Self-ONNs
Optimizer	Adam	Batch Size	16
Image Size	224	Stop Criteria	Loss
Maximum Number of Epochs	30	Epochs Patience	5
Maximum Epochs Stop	10	Learning Factor	0.2
Number of Folds	5	Learning Rate (LR)	0.0005
Standard Deviation (STD)	[0.4116, 0.3645, 0.2597]	Mean (M)	[0.2552, 0.4666, 0.8804]

**Table 4:** Classification results of all models for the RMB brain images. Bold represents the best-performing model.

Image Type	Name of the Network Model	Overall	Weighted			
		Accuracy (A)	Precession (P)	Recall (R)	Specificity (S)	F1 Score (F_s_)
RMB Images	Self-ONN4L1DN	93.90	93.48	93.77	94.34	93.50
	Self-ONN4L	93.76	93.56	93.76	94.28	93.75
	Self-ONN6L	94.19	94.85	94.29	95.47	94.58
	Self-ONN6L1DN	95.50	95.53	95.20	96.11	95.31
	Vanilla CNN8L	92.95	92.89	92.92	93.95	92.78
	Vanilla CNN6L	92.83	92.76	92.90	93.87	92.45
	DenseNet201	94.58	94.55	94.28	95.84	94.80
	ResNet50	95.89	95.94	95.29	96.81	95.16
	ResNet101	95.90	95.96	95.89	95.89	95.86
	MicrowaveBrainImage Network (MBINet)	96.97	96.93	96.85	97.95	96.83

**Table 5:** Quantitative assessments of all models. Bold represents the best-performing model.

Name of the Network Model	Accuracy (A)		Precession (P)		Recall (R)		Specificity (S)		F1 Score (F_s_)	
	M	STD	M	STD	M	STD	M	STD	M	STD
Self-ONN4L1DN	0.9390	0.0129	0.9348	0.0133	0.9377	0.0130	0.9434	0.0125	0.9350	0.0133
Self-ONN4L	0.9376	0.0130	0.9356	0.0132	0.9376	0.0130	0.9428	0.0125	0.9375	0.0131
Self-ONN6L	0.9419	0.0126	0.9485	0.0119	0.9429	0.0125	0.9547	0.0112	0.9458	0.0122
Self-ONN6L1DN	0.9550	0.0112	0.9553	0.0111	0.9520	0.0115	0.9611	0.0104	0.9531	0.0114
Vanilla CNN8L	0.9295	0.0138	0.9289	0.0139	0.9292	0.0138	0.9395	0.0129	0.9278	0.0140
Vanilla CNN6L	0.9283	0.0139	0.9276	0.0140	0.9290	0.0139	0.9387	0.0129	0.9245	0.0143
DenseNet201	0.9458	0.0122	0.9455	0.0122	0.9428	0.0125	0.9584	0.0108	0.9480	0.0120
ResNet50	0.9589	0.0107	0.9594	0.0106	0.9529	0.0114	0.9681	0.0095	0.9516	0.0116
ResNet101	0.9590	0.0107	0.9596	0.0106	0.9589	0.0107	0.9589	0.0107	0.9586	0.0107
MBINet	0.9697	0.0092	0.9693	0.0093	0.9685	0.0094	0.9795	0.0076	0.9683	0.0095

**Table 6:** Comparison results of the proposed model with the existing models. Bold represents the best-performing model.

Ref.	Year	Name of the Existing Models	Acc. (%)	Prc. (%)	Rec. (%)	Spec. (%)	F_s_ (%)	OCP (%)
[33]	2022	FT-DenseNet201	94.58	94.55	94.28	95.84	94.80	94.81
[34]	2020	PT-DenseNet201	94.66	94.62	94.76	94.68	93.95	94.53
[35]	2019	DP-DenseNet	93.10	93.88	94.09	94.87	94.98	94.18
[37]	2021	Differential-DCNN	95.80	95.73	95.62	95.61	95.11	95.57
[38]	2021	CMP-CNN	91.87	91.85	91.82	92.95	91.72	92.04
[39]	2021	ResNet-50	95.89	95.94	95.29	95.71	95.16	95.59
[40]	2022	Fine-tuned ResNet101	95.90	95.96	95.89	95.89	95.86	95.82
**Proposed**	**2023**	**MBINet**	**96.97**	**96.93**	**96.85**	**97.95**	**96.83**	**97.10**
